# *N*,*N*′-Bis[2-(methoxycarbonyl)ethyl]ethane-1,2-diammonium dichloride

**DOI:** 10.1107/S1600536808016565

**Published:** 2008-06-07

**Authors:** Goran N. Kaluderović, Anchan Paethanom, Christoph Wagner, Tibor J. Sabo, Harry Schmidt

**Affiliations:** a Institut für Chemie, Martin-Luther-Universität Halle-Wittenberg, Kurt-Mothes-Strasse 2, D-06120 Halle, Germany; b Faculty of Chemistry, University of Belgrade, Studentski trg 12-14, PO Box 158, 11000 Belgrade, Serbia

## Abstract

In the crystal structure of the title compound, C_10_H_22_N_2_O_4_^2+^·2Cl^−^ or (H_2_Me_2_eddp)Cl_2_ (H_2_Me_2_eddp^2+^ is the dimethyl *N*,*N*′-di-3-propane­carboxylato­ethane-1,2-diyldiimin­ium cation), the packing is stabilized by an infinite two-dimensional ⋯Cl⋯H—N—H⋯Cl⋯ hydrogen-bonding network. In addition, short C—H⋯Cl contacts are observed.

## Related literature

For related literature, see: Aakeröy *et al.* (1999 ▶); Bruhn *et al.* (2008 ▶); Kaluderović & Sabo (2002 ▶); Kaluderović *et al.* (2005 ▶, 2007 ▶, 2008 ▶); Krajčinović *et al.* (2008 ▶); Mijatović *et al.* (2005 ▶).
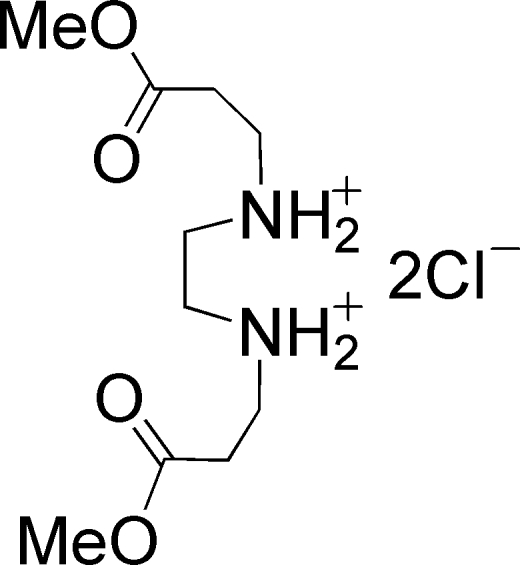


## Experimental

### 

#### Crystal data

C_10_H_22_N_2_O_4_^2+^·2Cl^−^
*M* *_r_* = 305.20
Monoclinic, 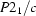

*a* = 8.9030 (8) Å
*b* = 10.3327 (10) Å
*c* = 8.3269 (10) Å
β = 101.763 (10)°
*V* = 749.93 (13) Å^3^
*Z* = 2
Mo *K*α radiation
μ = 0.44 mm^−1^
*T* = 293 (2) K
0.42 × 0.12 × 0.10 mm

#### Data collection

Stoe STADI4 diffractometer
Absorption correction: none
5296 measured reflections
1324 independent reflections
1021 reflections with *I* > 2σ(*I*)
*R*_int_ = 0.057
2 standard reflections frequency: 60 min intensity decay: random variation ±5%

#### Refinement

*R*[*F* ^2^ > 2σ(*F* ^2^)] = 0.036
*wR*(*F* ^2^) = 0.085
*S* = 1.13
1324 reflections
116 parameters
H atoms treated by a mixture of independent and constrained refinement
Δρ_max_ = 0.24 e Å^−3^
Δρ_min_ = −0.22 e Å^−3^

### 

Data collection: *STADI4* (Stoe & Cie, 1996 ▶); cell refinement: *STADI4*; data reduction: *STADI4*; program(s) used to solve structure: *SHELXS97* (Sheldrick, 2008 ▶); program(s) used to refine structure: *SHELXL97* (Sheldrick, 2008 ▶); molecular graphics: *DIAMOND* (Brandenburg, 2001 ▶); software used to prepare material for publication: *SHELXL97*.

## Supplementary Material

Crystal structure: contains datablocks I, global. DOI: 10.1107/S1600536808016565/pk2097sup1.cif

Structure factors: contains datablocks I. DOI: 10.1107/S1600536808016565/pk2097Isup2.hkl

Additional supplementary materials:  crystallographic information; 3D view; checkCIF report

## Figures and Tables

**Table 1:** Hydrogen-bond geometry (Å, °)

*D*—H⋯*A*	*D*—H	H⋯*A*	*D*⋯*A*	*D*—H⋯*A*
N—H3⋯Cl^i^	0.97 (3)	2.10 (3)	3.064 (2)	171 (2)
N—H4⋯Cl	0.85 (2)	2.30 (2)	3.092 (2)	156 (2)
C3—H8⋯Cl^ii^	0.95 (2)	2.73 (3)	3.619 (3)	156.3 (18)

Symmetry codes: (i) 
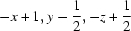
; (ii) 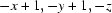.

**Fig. 1.**
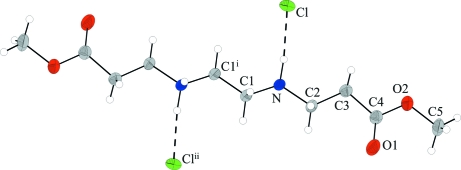
DIAMOND representation of (H2Me2eddp)Cl2. [Symmetry code: (i) -x + 1, -y + 1, -z + 1; (ii) 1 - x, y-1/2, -z + 1/2]

**Fig. 2.**
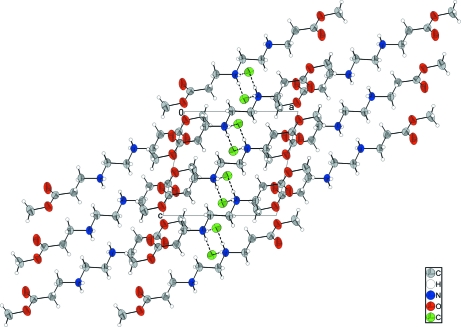
Network of H-bonding viewed along b-axis.

**Fig. 3.**
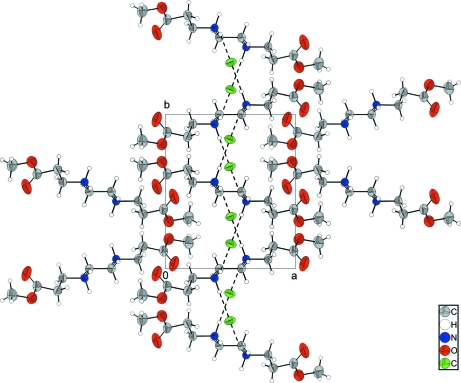
Network of H-bonding viewed along c-axis.

## supplementary crystallographic information

### Comment

The title compound (H_2_Me_2_eddp)Cl_2_ belongs to a class of compounds that have recently been used as ligand precursors in the synthesis of Co(III), Pt(II) and Pt(IV) complexes (Kaluđerović & Sabo, 2002; Kaluđerović *et al.*, 2008). The platinum complexes have been tested against various types of tumor cell lines and some of them have shown promising results in *in vitro* studies (Kaluđerović *et al.*, 2005; Mijatović *et al.*, 2005).  There are few crystal structures of these ligand precursors, or indeed of the corresponding platinum complexes, reported in the literature. To date, only four solid state structures of metal complexes containing platinum(IV) (Kaluđerović *et al.*, 2007, 2008; Krajčinović *et al.*, 2008), and only one crystal structure of ligand precursor *O,O'*-diisopropyl-ethylenediammonium-(*S,S*)-di-2-propanoate dichloride, [(*S*,*S*)-H_2_*i*-Pr_2_eddp]Cl_2_ (Krajčinović *et al.*, 2008) have been described.

Bond lengths and angles for the title compound are in the same range as found for [(*S*,*S*)-H_2_*i*-Pr_2_eddp]Cl_2_ (Krajčinović *et al.*, 2008). All non H atoms in the H_2_Me_2_eddp^2+^ cation are essentially co-planar with the largest deviation being for the C1 atom (0.175 (2) Å). The solid-state structure is stabilized by H-bonds. The H_2_Me_2_eddp^2+^ cations are joined in infinite two-dimensional networks through H-bonds *via* N—H groups and chloride anions (···Cl···H—N—H···Cl···; Figs. 2 and 3). The structural parameters of these two hydrogen bonds (N—H3···Cl = 3.064 (2) Å, N—H3···Cl = 171 (2)°, N—H4···Cl = 3.092 (2) Å, N—H4···Cl = 156 (2)°) are in accord with analogous hydrogen bonds in [(*S*,*S*)-H_2_*i*-Pr_2_eddp]Cl_2_ (Krajčinović *et al.*, 2008). Furthermore, short C—H···Cl contacts (C—H···Cl = 3.619 (3) Å, C—H···Cl = 156 (2)°) provide additional stabilization to the structure (Aakeröy *et al.*, 1999; Bruhn *et al.*, 2008).

### Experimental

The title compound was obtained as described in literature (Kaluđerović & Sabo, 2002). Colourless single crystals suitable for X-ray structure determination were obtained from mother liquor by slow evaporation at room temperature over several days.

### Refinement

The amine and methylene H atoms were found in a difference map and refined while methyl H atoms were positioned geometrically and treated as riding, with C–H bond lengths constrained to 0.96 Å.

### Crystal data

**Table:**

C_10_H_22_N_2_O_4_^2+^·2Cl^–^	*F*_000_ = 324
*M**_r_* = 305.20	*D*_x_ = 1.352 Mg m^−^^3^
Monoclinic, *P*2_1_/*c*	Mo *K*α radiation λ = 0.71073 Å
Hall symbol: -P 2ybc	Cell parameters from 26 reflections
*a* = 8.9030 (8) Å	θ = 7.7–12.2º
*b* = 10.3327 (10) Å	µ = 0.44 mm^−^^1^
*c* = 8.3269 (10) Å	*T* = 293 (2) K
β = 101.763 (10)º	Needle, colourless
*V* = 749.93 (13) Å^3^	0.42 × 0.12 × 0.10 mm
*Z* = 2	

### Data collection

**Table:**

Stoe STADI4 diffractometer	*R*_int_ = 0.057
Radiation source: fine-focus sealed tube	θ_max_ = 25.1º
Monochromator: graphite	θ_min_ = 2.3º
*T* = 293(2) K	*h* = −10→10
ω/2θ scans	*k* = −12→12
Absorption correction: none	*l* = −9→9
5296 measured reflections	2 standard reflections
1324 independent reflections	every 60 min
1021 reflections with *I* > 2σ(*I*)	intensity decay: random variation +−5%

### Refinement

**Table:**

Refinement on *F*^2^	Hydrogen site location: inferred from neighbouring sites
Least-squares matrix: full	H atoms treated by a mixture of independent and constrained refinement
*R*[*F*^2^ > 2σ(*F*^2^)] = 0.036	*w* = 1/[σ^2^(*F*_o_^2^) + (0.0263*P*)^2^ + 0.2601*P*] where *P* = (*F*_o_^2^ + 2*F*_c_^2^)/3
*wR*(*F*^2^) = 0.085	(Δ/σ)_max_ < 0.001
*S* = 1.13	Δρ_max_ = 0.24 e Å^−^^3^
1324 reflections	Δρ_min_ = −0.22 e Å^−^^3^
116 parameters	Extinction correction: SHELXL97 (Sheldrick, 2008), Fc^*^=kFc[1+0.001xFc^2^λ^3^/sin(2θ)]^-1/4^
Primary atom site location: structure-invariant direct methods	Extinction coefficient: 0.012 (3)
Secondary atom site location: difference Fourier map	

### Special details

**Table:**

Geometry. All e.s.d.'s (except the e.s.d. in the dihedral angle between two l.s. planes) are estimated using the full covariance matrix. The cell e.s.d.'s are taken into account individually in the estimation of e.s.d.'s in distances, angles and torsion angles; correlations between e.s.d.'s in cell parameters are only used when they are defined by crystal symmetry. An approximate (isotropic) treatment of cell e.s.d.'s is used for estimating e.s.d.'s involving l.s. planes.
Refinement. Refinement of *F*^2^ against ALL reflections. The weighted *R*-factor *wR* and goodness of fit *S* are based on *F*^2^, conventional *R*-factors *R* are based on *F*, with *F* set to zero for negative *F*^2^. The threshold expression of *F*^2^ > σ(*F*^2^) is used only for calculating *R*-factors(gt) *etc*. and is not relevant to the choice of reflections for refinement. *R*-factors based on *F*^2^ are statistically about twice as large as those based on *F*, and *R*- factors based on ALL data will be even larger.

### Fractional atomic coordinates and isotropic or equivalent isotropic displacement parameters (Å^2^)

**Table:**

	*x*	*y*	*z*	*U*_iso_*/*U*_eq_	
C1	0.5853 (3)	0.4998 (2)	0.5051 (3)	0.0387 (6)	
H2	0.624 (3)	0.586 (2)	0.507 (3)	0.046 (7)*	
H1	0.636 (3)	0.454 (2)	0.596 (3)	0.051 (7)*	
C2	0.7898 (3)	0.4385 (3)	0.3579 (3)	0.0401 (6)	
H5	0.835 (3)	0.409 (2)	0.462 (3)	0.052 (8)*	
H6	0.818 (3)	0.525 (3)	0.344 (3)	0.046 (7)*	
C3	0.8277 (3)	0.3565 (3)	0.2221 (3)	0.0421 (6)	
H8	0.756 (3)	0.376 (2)	0.126 (3)	0.047 (7)*	
H7	0.819 (3)	0.272 (2)	0.248 (3)	0.045 (7)*	
C4	0.9857 (3)	0.3853 (2)	0.1953 (3)	0.0417 (6)	
C5	1.1771 (3)	0.3294 (3)	0.0491 (3)	0.0609 (8)	
H9	1.1942	0.4207	0.0408	0.091*	
H11	1.1832	0.2882	−0.0528	0.091*	
H10	1.2539	0.2937	0.1356	0.091*	
N	0.6217 (2)	0.43799 (19)	0.3563 (3)	0.0345 (5)	
H3	0.585 (3)	0.349 (3)	0.352 (3)	0.051 (7)*	
H4	0.577 (3)	0.480 (2)	0.271 (3)	0.047 (8)*	
O1	1.0669 (2)	0.4681 (2)	0.2636 (3)	0.0799 (7)	
O2	1.02593 (19)	0.30760 (17)	0.0849 (2)	0.0566 (5)	
Cl	0.49596 (7)	0.66139 (5)	0.11984 (7)	0.0475 (2)	

### Atomic displacement parameters (Å^2^)

**Table:**

	*U*^11^	*U*^22^	*U*^33^	*U*^12^	*U*^13^	*U*^23^
C1	0.0311 (12)	0.0375 (13)	0.0491 (15)	−0.0017 (10)	0.0122 (11)	−0.0005 (11)
C2	0.0277 (12)	0.0450 (15)	0.0488 (15)	−0.0014 (10)	0.0104 (11)	−0.0051 (12)
C3	0.0363 (13)	0.0453 (16)	0.0444 (14)	−0.0044 (11)	0.0076 (11)	−0.0059 (12)
C4	0.0340 (13)	0.0505 (14)	0.0390 (13)	0.0011 (11)	0.0038 (10)	−0.0028 (11)
C5	0.0503 (16)	0.0728 (19)	0.0679 (18)	0.0073 (14)	0.0312 (14)	−0.0037 (16)
N	0.0272 (10)	0.0305 (10)	0.0461 (12)	0.0013 (8)	0.0080 (8)	0.0056 (9)
O1	0.0440 (11)	0.1084 (17)	0.0928 (16)	−0.0259 (12)	0.0270 (11)	−0.0537 (14)
O2	0.0499 (11)	0.0618 (12)	0.0648 (12)	−0.0047 (9)	0.0271 (9)	−0.0190 (9)
Cl	0.0542 (4)	0.0336 (3)	0.0529 (4)	0.0092 (3)	0.0064 (3)	0.0026 (3)

### Geometric parameters (Å, °)

**Table:**

C1—N	1.487 (3)	C3—H7	0.91 (2)
C1—C1^i^	1.505 (4)	C4—O1	1.188 (3)
C1—H2	0.95 (2)	C4—O2	1.324 (3)
C1—H1	0.93 (3)	C5—O2	1.454 (3)
C2—N	1.494 (3)	C5—H9	0.9600
C2—C3	1.506 (3)	C5—H11	0.9600
C2—H5	0.93 (2)	C5—H10	0.9600
C2—H6	0.94 (3)	N—H3	0.97 (3)
C3—C4	1.498 (3)	N—H4	0.85 (3)
C3—H8	0.94 (2)		
			
N—C1—C1^i^	110.0 (3)	H8—C3—H7	109 (2)
N—C1—H2	105.7 (14)	O1—C4—O2	123.0 (2)
C1^i^—C1—H2	111.0 (14)	O1—C4—C3	124.8 (2)
N—C1—H1	107.8 (15)	O2—C4—C3	112.2 (2)
C1^i^—C1—H1	111.5 (15)	O2—C5—H9	109.5
H2—C1—H1	111 (2)	O2—C5—H11	109.5
N—C2—C3	111.63 (19)	H9—C5—H11	109.5
N—C2—H5	104.7 (15)	O2—C5—H10	109.5
C3—C2—H5	113.1 (16)	H9—C5—H10	109.5
N—C2—H6	107.3 (15)	H11—C5—H10	109.5
C3—C2—H6	109.6 (15)	C1—N—C2	112.25 (18)
H5—C2—H6	110 (2)	C1—N—H3	108.0 (14)
C4—C3—C2	111.1 (2)	C2—N—H3	109.2 (14)
C4—C3—H8	108.8 (14)	C1—N—H4	109.2 (17)
C2—C3—H8	107.9 (15)	C2—N—H4	107.4 (16)
C4—C3—H7	110.7 (15)	H3—N—H4	111 (2)
C2—C3—H7	109.0 (15)	C4—O2—C5	116.20 (19)
			
N—C2—C3—C4	164.5 (2)	C3—C2—N—C1	170.4 (2)
C2—C3—C4—O1	−5.0 (4)	O1—C4—O2—C5	−0.5 (4)
C2—C3—C4—O2	175.3 (2)	C3—C4—O2—C5	179.2 (2)
C1^i^—C1—N—C2	178.3 (3)		

Symmetry codes: (i) −*x*+1, −*y*+1, −*z*+1.

### Hydrogen-bond geometry (Å, °)

**Table:**

*D*—H···*A*	*D*—H	H···*A*	*D*···*A*	*D*—H···*A*
N—H3···Cl^ii^	0.97 (3)	2.10 (3)	3.064 (2)	171 (2)
N—H4···Cl	0.85 (2)	2.30 (2)	3.092 (2)	156 (2)
C3—H8···Cl^iii^	0.95 (2)	2.73 (3)	3.619 (3)	156.3 (18)

Symmetry codes: (ii) −*x*+1, *y*−1/2, −*z*+1/2; (iii) −*x*+1, −*y*+1, −*z*.
